# Targeting Hippo Signaling Pathway with a Boron Derivative, Sodium Pentaborate Pentahydrate (NaB): Therapeutic Strategies in Colorectal Cancer

**DOI:** 10.3390/ph18081171

**Published:** 2025-08-08

**Authors:** Büşra Yüksel, Fikrettin Şahin, Nezaket Türkel

**Affiliations:** Department of Genetics and Bioengineering, Faculty of Engineering, Yeditepe University, Kayışdağı, Istanbul 34755, Turkey; busra.yuksel@yeditepe.edu.tr (B.Y.); fsahin@yeditepe.edu.tr (F.Ş.)

**Keywords:** sodium pentaborate pentahydrate, colorectal cancer, apoptosis, cell cycle arrest, Hippo pathway

## Abstract

**Background/Objectives**: Colorectal cancer (CRC) remains a leading cause of cancer-related mortality globally, highlighting the urgent need for novel therapeutic strategies. This study aimed to investigate the anticancer potential of sodium pentaborate pentahydrate (NaB) in CRC by evaluating its effects on human colorectal cancer cell lines and elucidating underlying molecular mechanisms. **Methods**: The cytotoxic and molecular effects of NaB were assessed in three human CRC cell lines (HCT-116, HT-29, and COLO-205) and one normal colon epithelial cell line (CCD-18CO). Cell viability assays were conducted to determine time- and dose-dependent responses. Apoptosis, cell cycle progression, colony formation, and migration capacity were evaluated. Gene and protein expression analyses were performed to examine apoptosis-related, DNA damage response, cell cycle, and Hippo signaling pathway components. **Results**: NaB significantly reduced cancer cell viability in a time- and dose-dependent manner, with minimal cytotoxicity to normal colon cells. It induced marked apoptosis, especially in HCT-116 and COLO-205 cells, and caused G2/M cell cycle arrest. In HCT-116 cells, NaB suppressed proliferation by downregulating PCNA and MKI-67 and reduced colony formation and migration. Molecular analyses revealed upregulation of pro-apoptotic BAX and downregulation of BCL-2, ATM, ATR, and cell cycle–related genes. NaB also inhibited oncogenic Hippo signaling by enhancing YAP1 phosphorylation and decreasing CTGF and CYR61 expression. **Conclusions**: These findings demonstrate that sodium pentaborate pentahydrate exerts selective anticancer effects on colorectal cancer cells through the induction of apoptosis, cell cycle arrest, and suppression of key oncogenic pathways. NaB represents a promising candidate for further development as a therapeutic agent in CRC treatment.

## 1. Introduction

Colorectal cancer (CRC) is the third most common malignant tumor worldwide and the second leading cause of cancer-related deaths [1]. Risk factors such as low-fiber, high-fat diets, obesity, and sedentary lifestyles are known to promote the development of CRC. Current treatment strategies for CRC include radiotherapy, chemotherapy, and surgical resection [2]. However, chemotherapy drugs have significant disadvantages, including cytotoxic effects, lack of tumor specificity, and the development of drug resistance. Although radiotherapy shows promise for treating colon cancer, the high radiation intensity can damage vital organs. Therefore, despite these treatments, current therapeutic strategies remain limited, underscoring the urgent need to identify new therapeutic targets and driver pathways in CRC.

Boron, a nonmetallic element found in Group 13 (IIIA) of the periodic table, is an essential micronutrient for humans, animals, and plants [3,4]. Boron compounds possess a unique ability to switch between neutral trigonal planar sp2 and tetrahedral sp3 hybridization states, allowing them to form coordinate covalent bonds. This property makes boron a promising candidate for the development of boron-containing drugs [5,6]. In 2003, Bortezomib (Velcade) became the first boronic acid-containing drug approved by the U.S. Food and Drug Administration (FDA) for the treatment of multiple myeloma [7]. Other FDA-approved boron-containing drugs include Tavaborate, Ixazomib, Crisaborole, and Vaborbactam [8].

Boron has been implicated in the treatment of several cancers, including breast [9], glioblastoma [10], lung [11], prostate [12], and hepatocellular carcinoma [13]. Sodium pentaborate pentahydrate (NaB, NaB_5_O_8_·5H_2_O), a boron derivative, has demonstrated efficacy in various studies for treating obesity [14], promoting wound healing [15], and combating cancer [16]. A study by Mohammed et al. reported that NaB suppressed cell proliferation, induced apoptosis, and increased YAP and PD-L1 expression in breast cancer [9]. Additionally, NaB has been shown to upregulate apoptotic gene expression in non-small cell lung cancer [11]. Recent studies suggest that NaB may have significant anti-tumorigenic effects in colon cancer by targeting the Hippo signaling pathway.

The Hippo signaling pathway was first discovered in Drosophila Melanogaster, where it plays a crucial role in the formation of eye and wing discs [17]. This pathway includes several core components such as mammalian STE20-like kinase 1/2 (MST1/2), protein Salvador homologue 1 (SAV1), large tumor suppressor kinase 1/2 (LATS1/2), MOBKL1A/B (MOB1A/B), Yes-associated protein 1 (YAP), WW-domain-containing transcription regulator 1 (TAZ), and the transcriptional enhanced associated domain (TEAD) family [18]. YAP/TAZ are transcriptional coactivators that interact with TEAD, and overexpression of YAP can result in increased tissue proliferation and organ growth [19].

Recent studies have established a strong correlation between elevated YAP1 activity and poor clinical outcomes in colorectal cancer (CRC) patients. As the main effector of the Hippo signaling pathway, YAP1 promotes tumorigenesis when aberrantly activated, driving uncontrolled cell proliferation, resistance to apoptosis, epithelial–mesenchymal transition (EMT), and metastasis. In CRC, nuclear accumulation of unphosphorylated YAP1 has been associated with advanced tumor stage, lymph node metastasis, and resistance to chemotherapy, underscoring its clinical relevance. Furthermore, YAP1 target genes such as CTGF and CYR61 contribute to extracellular matrix remodeling and angiogenesis, thereby facilitating tumor progression [20,21].

In light of this, we aimed to investigate the anticancer effects of NaB in CRC cell models, with particular attention to its impact on key molecular pathways such as apoptosis, DNA damage response, and Hippo signaling modulation. Our findings provide novel insights into the mechanistic actions of NaB and highlight its potential as a therapeutic agent in CRC treatment.

Based on this framework, we proposed that sodium pentaborate pentahydrate (NaB) holds significant potential to extend survival and reduce side effects compared to conventional cancer therapies, particularly in the treatment of colon cancer. This study investigates the impact of NaB on apoptosis, cell cycle regulation, and cell proliferation in the HCT-116, Colo-205, and HT-29 colon cancer cell lines in vivo. Furthermore, we explore the modulation of the Hippo signaling pathway by NaB, positioning it as a promising anti-tumorigenic strategy in colorectal cancer.

## 2. Results

### 2.1. Exploring the Effects of NaB Treatment on Cell Survival

**Figure 1:**
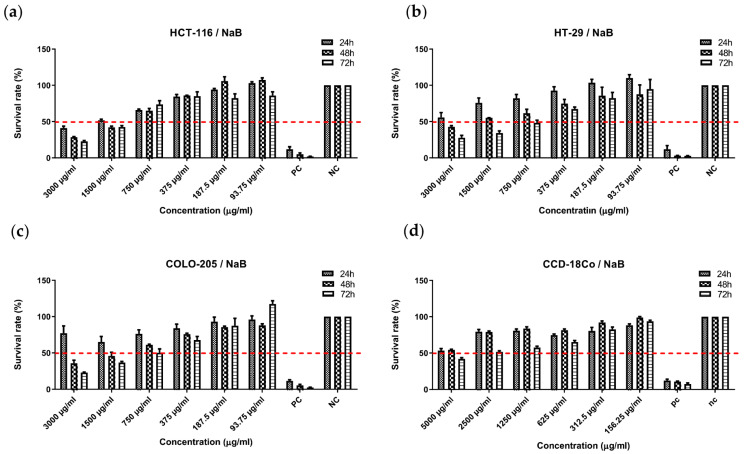
Cell survival rates (%) of (**a**) HCT-116, (**b**) HT-29, (**c**) COLO-205 cancer cell lines, and (**d**) CCD-18Co normal cell line at 24th, 48th, and 72nd post-treatment with NaB (sodium pentaborate pentahydrate). Data are presented as the percentage of viable cells relative to the negative control at each time point, highlighting the differential effects of NaB on cancer and normal cells.

The anticancer potential of NaB was examined in HCT-116, COLO-205, HT-29, and CCD-18C0 cell lines using MTS assay. Treatment reduced cell survival in a time—(24,48, and 72 h) and concentration-dependent (93.75–3000 µg/mL) manner for HCT-116, COLO-205, and HT-29 cancer cell lines (Figure 1a–c). Also, NaB (5000–156.25 µg/mL) was used to treat CCD-18CO normal colon cell lines (Figure 1d). The half-maximal inhibitory concentration (IC50) was calculated and is shown in Table 1. The IC50 value of NaB was 1000 µg/mL for the HCT-116 cancer cell line, 500 µg/mL for the HT-29 and COLO-205 cancer cell lines, and 2500 µg/mL for the CCD-18C0 normal colon cell line. The obtained results strongly indicated that NaB exhibited a significant anticancer effect across all tested cancer cell lines, including HCT-116, COLO-205, and HT-29, when compared to the normal colon cell line, CCD-18Co. These findings demonstrate that NaB enhances its cytotoxic effects in cancer cells while having the potential to protect healthy colon cells. This selective effect may have the capacity to minimize the adverse effects commonly associated with conventional chemotherapeutic agents, making NaB a promising candidate for further investigation in targeted cancer therapies.

### 2.2. NaB-Induced Apoptosis and Caspase Activation in HCT-116, HT-29, and COLO-205 Colorectal Cancer Cell Lines

**Figure 2:**
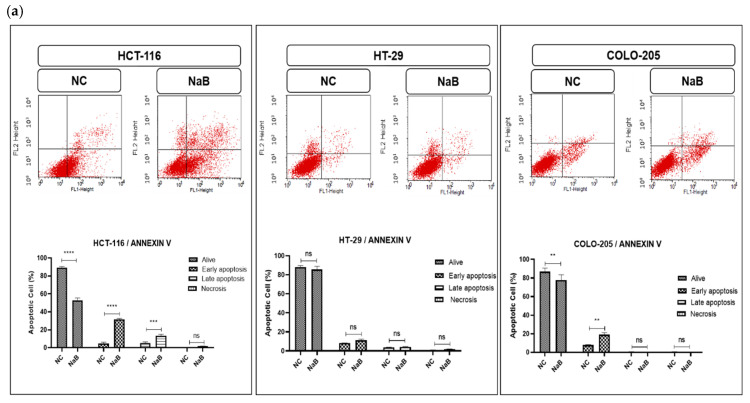

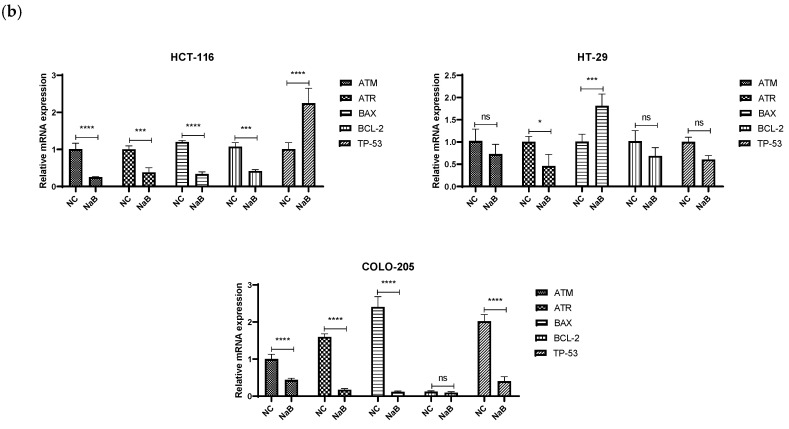
(**a**) Representative Annexin V-FITC/PI staining results for HCT-116, HT-29, and COLO-205 cancer cells at 72 h with quantitative analysis; values are presented as mean ± SD of three independent experiments. (**b**) Representative graph of apoptotic and anti-apoptotic gene expression profiles of HCT-116, HT-29, and COLO-205 cells after 72 h. (ns: non-significant, *: *p* < 0.05, **: *p* < 0.01, ***: *p* < 0.001, ****: *p* < 0.0001).

The effects of NaB on apoptosis activity (Figure 2a) were determined in HCT-116, HT-29, and COLO-205 cancer cell lines by flow cytometry, Real-time PCR, and caspase activity assay. All assays were performed using IC-50 values of NaB concentration according to MTS assay results after 72 h. Annexin-V data showed no significant difference on apoptosis in NaB-treated HT-29 cancer cells compared to the negative control (Figure 2a). Moreover, NaB significantly increased early apoptosis (27.26%) and late apoptosis (8.15%) in HCT-116 cancer cell lines (Figure 2a). The early apoptosis population also increased from 7.96% negative control cells to 19.04% with NaB-treated COLO-205 cancer cell lines (Figure 2a). Our results show that NaB treatment increased apoptosis in HCT-116 and COLO-205 cancer cell lines.

To confirm that the NaB induces the apoptosis mechanism, mRNA expression levels of important apoptotic genes were analyzed. The relative expression level of the pro-apoptotic BAX gene was elevated in the NaB-treated compared to the negative control (Figure 2b) in HCT-116 and COLO-205 cancer cell lines. This revealed that treating HCT-116 cells with NaB increased the mRNA expression level of TP-53 (≈1.23 fold), while levels of BCL-2 (≈0.41), ATM (≈0.25), and ATR (≈0.38) were significantly decreased (Figure 2). Moreover, there was no significant change in the levels of ATM, BCL-2, and TP-53 genes, but there were BAX (≈0.8 fold) genes significantly up-regulated when compared the negative control in HT-29 cancer cell lines (Figure 2b). Also, ATM, ATR, and TP-53 genes were downregulated with NaB treatment in COLO-205 cancer cell lines (Figure 2b).

**Figure 3:**
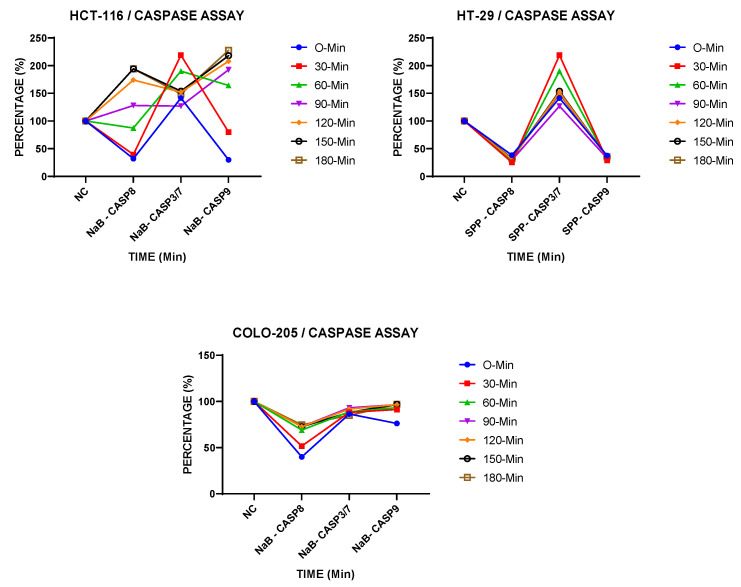
Caspase 8, caspase 3/7, and caspase 9 activation detected after treatment with IC-50 values of NaB in HCT-116, HT-29 in COLO-205 cancer cell line.

To demonstrate the activity of caspases, we measured Caspase-8, Caspase-3/7, and Caspase-9 activity using the Caspase-Glo 8, 3/7, and 9 assay kit. As shown in Figure 3, Caspase 3/7 and Caspase-8 activities were increased from 0 min to 150 min in NaB treatment conditions in HCT-116 cancer cell lines. In HT-29 cancer cell lines, Caspase-3/7 activity increased, while Caspase-8 and Caspase-9 activity decreased compared to the negative control (Figure 3). On the other hand, there were no significant differences in caspase activity in COLO-205 cancer cell lines.

### 2.3. Mechanisms of NaB-Induced Growth Inhibition in HCT-116, HT-29, and COLO-205 Colorectal Cancer Cell Lines

**Figure 4:**
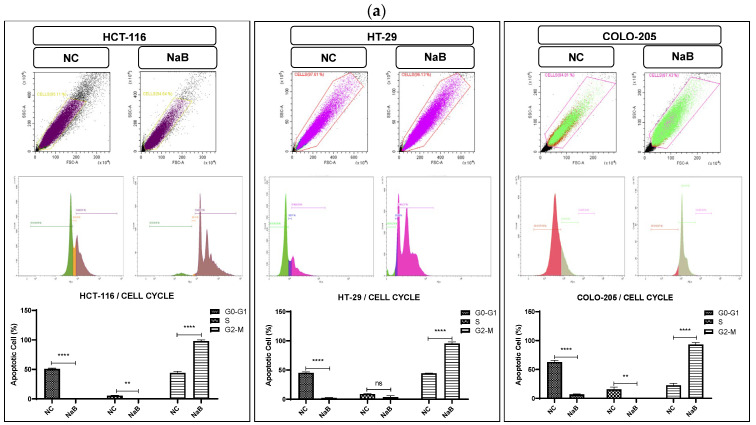

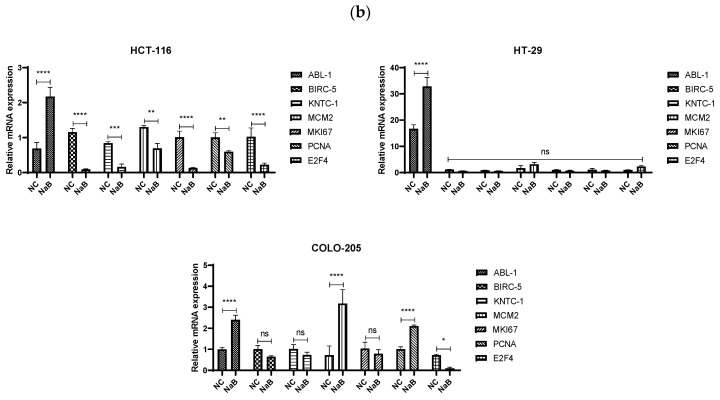
Distribution of cell cycle phases by flow cytometry for HCT-116, HT-29, and COLO-205 cells. (**a**) Distribution of cell cycle phases; the initial, middle, and last peaks show G0/G1, S, and G2-M, respectively, and the result of cell cycle analysis percentage of HCT-116 cells in G0/G1, S, and G2 phase compared with negative control. (**b**) Representative graph of cell cycle genes expression profiles of HCT-116, HT-29, and COLO-205 cells after 72 h. (ns: non-significant, *: *p* < 0.05, **: *p* < 0.01, ***: *p* < 0.001, ****: *p* < 0.0001).

To elucidate the mechanisms involved in inhibiting the growth of colon cancer cells, the cell cycle, Edu, colony-formation assay, scratch assay, and related mRNA expressions were performed. Comparing the cell proportions at each phase, no significant difference was detected in the G0/G1 and S phase treated with NaB (Figure 4a). Following analysis, NaB treatment surprisingly caused a significant increase in the percentage of cells in the G2/M phase in HCT-116 (from 43.9% to 97.9%), HT-29 (from 44.4% to 93.99%), and COLO-205 (from 22.58% to 93.06%).

Figure 4b shows the results of mRNA expression of cell cycle-regulation genes (ABL-1, BIRC-5, KNTC-1, MCM2, MKI-67, PCNA, and E2F4) related to NaB-treated HCT-116, HT-29, and COLO-205 cancer cell lines. The results of mRNA expression show that ABL-1 genes were significantly increased in all cancer cell lines. Moreover, BIRC-5, KNTC-1, MCM2, MKI-67, PCNA, and E2F4 genes were downregulated in HCT-116 cancer cell lines, and there was no significant change in the expression of the same genes in HT-29 cancer cell lines. Additionally, the expression of MCM2, PNCA, and E2F4 genes was reduced in COLO-205 cancer cell lines. 

**Figure 5:**
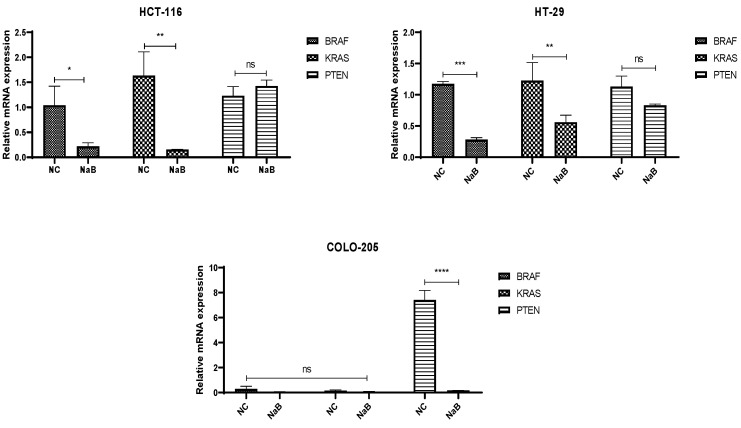
Representative graph of BRAF, KRAS, and PTEN genes expression profiles of HCT-116, HT-29, and COLO-205 cells after IC-50 values of NaB treatment 72 h. (ns: non-significant, *: *p* < 0.05, **: *p* < 0.01, ***: *p* < 0.001, ****: *p* < 0.0001).

qPCR analysis was performed to assess the expression levels of KRAS, BRAF, and PTEN genes in HCT116, HT29, and COLO205 colorectal cancer cell lines after NaB treatment. A notable downregulation of KRAS and BRAF expression was observed in both HCT116 and HT29 cells compared to untreated controls. Interestingly, in COLO205 cells, no significant change was detected in KRAS or BRAF expression levels, while PTEN expression was markedly decreased following treatment (Figure 5).

**Figure 6:**
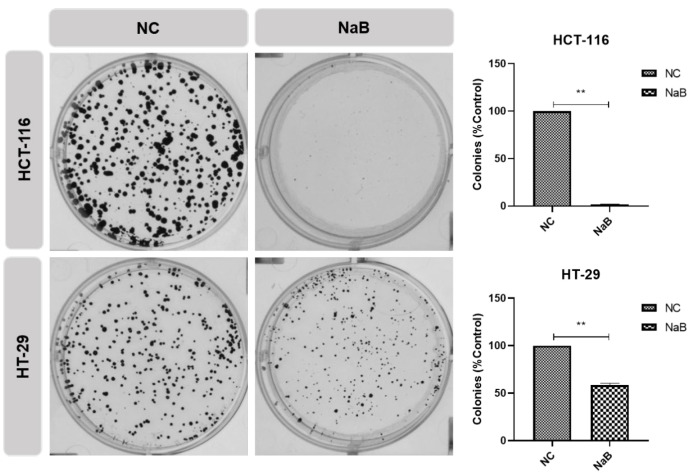
Cell colony formation assay. HCT-116 and HT-29 cancer cell lines were treated with the IC-50 value of NaB or control medium. (**: *p* < 0.01).

**Figure 7:**
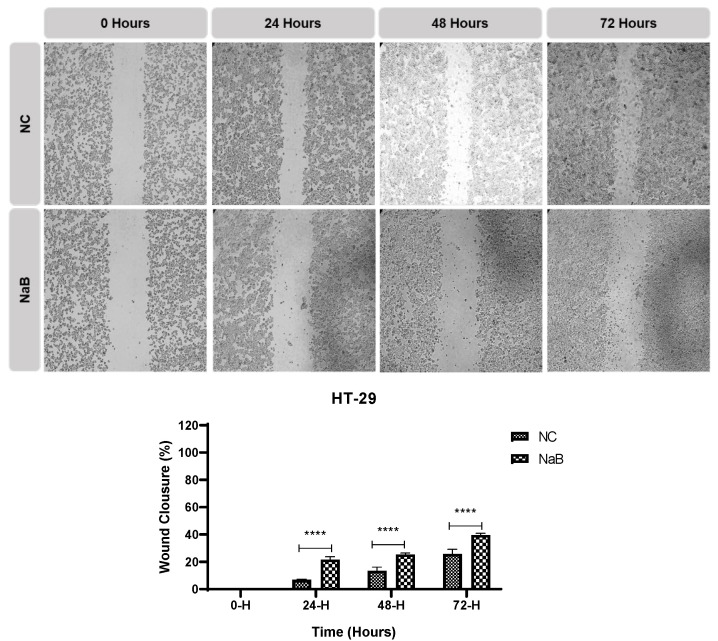
Closure of the gap created by scratch in HT-29 cancer cell line at 0 h, 24 h, 48 h, and 72 h timepoints. Quantification of the distance closed by the two cell lines (5× magnification, ****: *p* < 0.0001).

To quantify differences in growth characteristics, a colony-formation assay (CFA) was performed to examine how NaB treatment affected colony-forming capacities. The results showed that the HCT-116 cell line treated with NaB did not have colony-forming capacity (Figure 6). On the other hand, in the HT-29 cell line, the colony-forming capacity was reduced by half when NaB was applied (Figure 7). 

**Figure 8:**
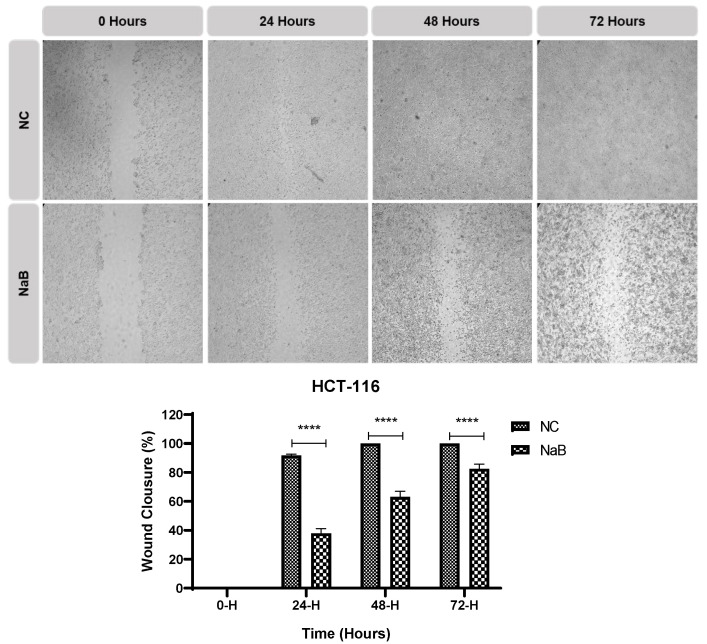
Closure of the gap created by scratch in HCT-116 cancer cell line at 0 h, 24 h, 48 h, and 72 h timepoints. Quantification of the distance closed by the two cell lines (5× magnification, ****: *p* < 0.0001).

**Figure 9:**
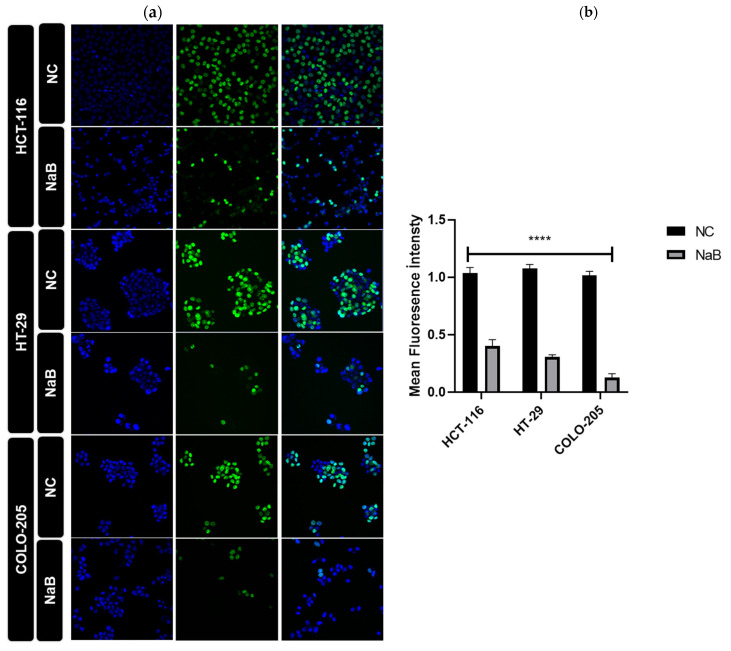
EdU DNA proliferation assay of HCT-116, HT-29, and COLO-205 cancer cell lines. Each cell line was treated with the IC-50 value of NaB. (**a**) The left image shows proliferating cells labeled with EdU-green channel. (DAPI-blue channel). (**b**) The graph shows fluorescence intensity, which was quantified using the Image J 1.54g program. (10× magnification, ns: non-significant, ****: *p* < 0.0001).

A scratch and EdU assay was performed to understand the effects of the applied NaB on the migratory ability and proliferation status of the cells. The results obtained from this in-cell migration assay showed that the migratory ability of NaB-treated HCT-116 and HT-29 cells was reduced (Figure 7 and Figure 8). According to the EdU assay, NaB treatment significantly hampered cell proliferation in HCT-116, HT-29, and COLO-205 cancer cell lines (Figure 9).

### 2.4. NaB Treatment Modulates Hippo Signaling Pathway and Expression of YAP1 Target Genes in HCT-116 and HT-29 Colorectal Cancer Cell Lines

**Figure 10:**
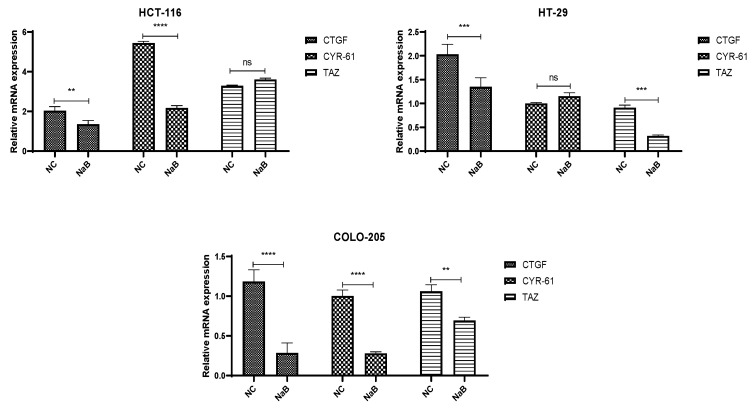
Representative graph of Hippo pathway component TAZ and its downstream transcriptional targets; CTGF and CYR-61 genes expression profiles of HCT-116, HT-29, and COLO-205 cells after IC-50 NaB treatment 72 h. (ns: non-significant, **: *p* < 0.01, ***: *p* < 0.001, ****: *p* < 0.0001).

NaB treatment led to a significant increase in the phosphorylation of YAP1, which plays a crucial role in the regulation of gene expression within the Hippo signaling pathway. In contrast, the mRNA expression levels of CTGF and CYR-61, two key target genes of YAP, were significantly decreased in the HCT-116 cancer cell line (Figure 10). This suggests that NaB treatment may inhibit the activation of YAP target genes, potentially impairing YAP’s oncogenic activity.

**Figure 11:**
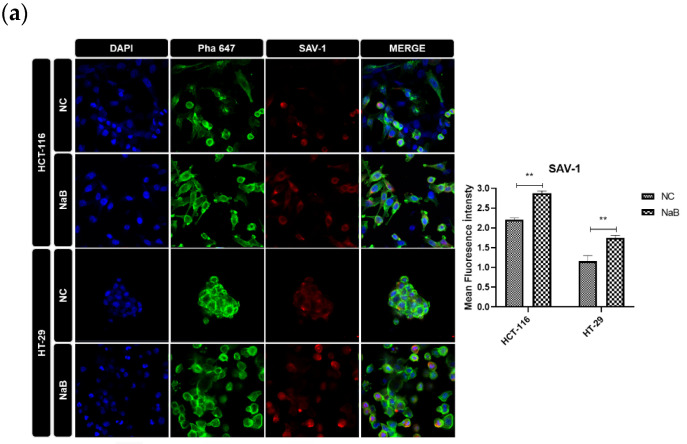

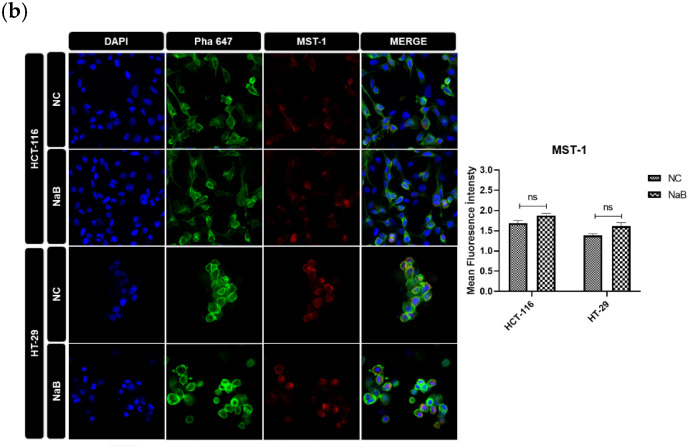

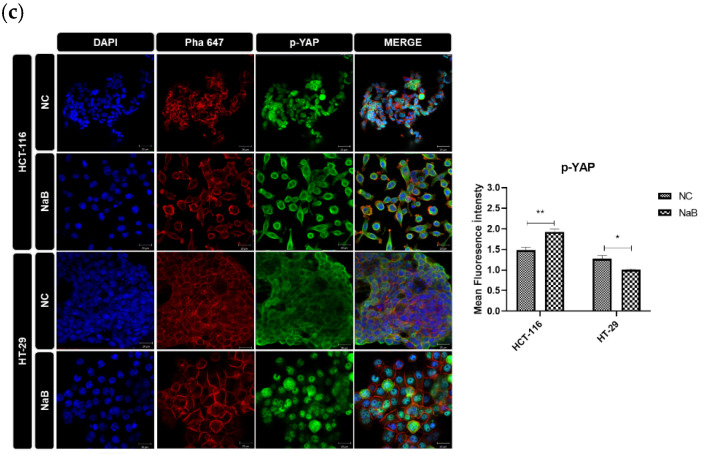
Representative immunofluorescence images showing (**a**) SAV-1, (**b**) MST-1, and (**c**) p-YAP staining in HCT-116 and HT-29 cancer cell lines. Magnification: 20×. Quantitative representation of the SAV-1, MST-1, and p-YAP fluorescence signals (ns: non-significant, *: *p* < 0.05, **: *p* < 0.01).

Additionally, NaB treatment resulted in a marked elevation in the expression of SAV1, a tumor-suppressor gene involved in the Hippo pathway. However, no significant differences were observed in the expression of MST-1 protein in either the HCT-116 or HT-29 cancer cell lines (Figure 11). This indicates that NaB may specifically affect certain components of the Hippo pathway, particularly those involved in tumor suppression, without altering MST-1 levels. Furthermore, NaB treatment led to a significant reduction in the mRNA expression of CTGF, CYR-61, and TAZ genes in the HCT-116 cells. This suggests that NaB might suppress the YAP-driven gene expression in these cells. On the other hand, CYR-61 expression in HT-29 cells did not show significant changes when compared to the negative control, highlighting a cell line-specific response to NaB treatment.

Overall, our results indicate that NaB treatment exerts notable effects on the Hippo signaling pathway, especially by modulating the phosphorylation of YAP1 and the expression of its downstream target genes. These effects appear to be cell-line dependent, with NaB showing distinct impacts in HCT-116 and HT-29 cells.

## 3. Discussion

Colon cancer remains a leading cause of cancer-related mortality worldwide, with standard treatments—surgical resection, chemotherapy, and radiotherapy—often resulting in limited efficacy and substantial adverse effects. Consequently, identifying novel and less toxic therapeutic strategies remains a pressing priority. Among emerging approaches, boron-based compounds such as sodium pentaborate pentahydrate (NaB) have garnered attention for their capacity to target multiple oncogenic pathways in colorectal cancer, offering a promising avenue for improved clinical outcomes [22].

In this study, we demonstrated that NaB exerts potent anticancer effects in human colorectal cancer cell lines while sparing normal colon cells, suggesting a substantial therapeutic window. The variations in IC_50_ values across cell lines may be attributed to distinct genetic backgrounds—particularly differences in p53 status (wild-type in HCT-116, mutant in HT-29 and COLO-205) [23]—and highlights the cell-specific nature of NaB’s efficacy. Although COLO-205 cells were employed in many of our experiments, their semi-suspension growth characteristics precluded their use in certain assays (including cell migration and colony-formation assays). Notably, COLO-205 shares a mutational profile similar to HT-29, as both cell lines are wild-type for KRAS and PTEN and harbor mutations in TP53 (different mutations) [24]. This genetic parallel may partly explain similarities in their response to specific treatments while highlighting the potential influence of shared molecular drivers on therapeutic outcomes.

NaB induced G2/M cell cycle arrest, downregulated cell cycle–associated genes (e.g., KNTC1, MCM2, MKI-67, PCNA, and E2F4), and increased ABL-1 expression. This reflects the distribution of mitotic progression, potentially pushing cells towards apoptosis. Importantly, colony formation and DNA synthesis (EdU incorporation) were significantly impaired, suggesting long-term suppression of proliferative capacity. Moreover, NaB attenuated migration in adherent lines (HCT-116 and HT-29), indicating its potential to impair tumor invasiveness.

NaB-induced apoptosis was validated by flow cytometry and caspase activation. Notably, HCT-116 exhibited strong Caspase 3/7 and Caspase 8 responses, consistent with both intrinsic and extrinsic apoptotic mechanisms. In contrast, HT-29 cells demonstrated a more modest response, potentially attributable to their mutant p53 profile. Alterations in BaX, Bcl2, ATM, ATR, and tp53 expression reinforces the idea that p53 functionality shapes apoptotic sensitivity to NaB.

A critical mechanistic insight of this study involves the modulation of the Hippo signaling pathway, often dysregulated in colorectal cancer [20]. NaB increased YAP1 phosphorylation and decreased the transcription of CTGF and CYR-61 in HCT-116 cells, along with upregulation of SAV1 and downregulation of TAZ, suggesting partial reactivation of Hippo tumor suppressor functions. However, we acknowledge that our conclusions about Hippo modulation are based on mRNA and protein expression, lacking functional validation. Future studies should incorporate YAP/TAZ knockdown or TEAD-reporter assays to directly test the pathway’s functional outcomes.

Although our in vitro findings on NaB suggest a favorable therapeutic window, in vivo studies are needed to evaluate tissue-specific accumulation, long-term safety, and optimal delivery methods. Previous studies on boron-based compounds have reported low systemic toxicity, but NaB-specific data remain limited and should be expanded through well-designed preclinical assessments.

## 4. Materials and Methods

### 4.1. Cell Lines and Cell Culture Conditions

HT-29 (HTB-38, human colorectal adenocarcinoma cell lines), Colo-205 (CCL-222, human adenocarcinoma cell lines), and HCT-116 (CRL-247, human colorectal adenocarcinoma cell lines) were obtained from the American Type Culture Collection (ATCC, Rockville, MD, USA). HT-29 cell lines were cultured in Roswell Park Memorial Institute medium (RPMI, #11875093, Invitrogen, Gibco, London, UK). HCT-116 and Colo-205 cell lines were cultured in Dulbecco’s Modified Eagle’s Medium (DMEM, #41966-029, Invitrogen, Gibco, London, UK). Each medium was supplemented with %1 Penicillin/Streptomycin/Amphotericin (PSA, Invitrogen, Gibco, London, UK) and %10 fetal bovine serum (FBS, #10500-064, Invitrogen, Gibco, London, UK) at 37 °C in a 5% CO_2_ incubator.

### 4.2. Cell Viability Assay

Sodium pentaborate pentahydrate (NaB, National Boron Research Institute-BOREN, Ankara, Turkey) cytotoxicity was measured through the MTS assay (3-(4,5-dimethyl-thiazol-2)-5-(3-carboxy-methoxy-phenyl)-2-(4-sulfo-phenyl)-2H-tetrazolium salt (MTS) (#G3582, CellTiter96 AqueousOne Solution; Promega, Southampton, UK) according to the manufacturer’s instructions. HCT-116, HT-29, and Colo-205 cells were seeded at the density of 5000 cells/well in a 96-well plate and incubated for 24 h. Then, NaB was added at six different concentrations (doses ranging from 3000 µg/mL to 93.75 µg/mL), and cells were further incubated for 24 h, 48 h, and 72 h. After incubation, NaB-containing medium was removed and an MTS solution (PBS solution included %10 MTS and 4.5 g/L D-glucose solution) was added followed by 90 min of incubation at 37 °C. Then, their absorbance was measured at 490 nm by using an ELISA plate reader (Biotek, Winooski, VT, USA). Cell survival data were reported as % of negative control (untreated cells) for each cell line.

### 4.3. Annexin V Assay

To apply the Annexin-V assay, HCT-116, HT-29, and COLO-205 cells were seeded in proper densities (50 × 103 cells/T25 flask) for treatment of NaB and negative control. After 24 h, the media were changed with NaB-containing media according to defined IC50 concentrations. After 72 h of treatment, cells Annexin V assay was performed according to manufacturer’s protocol (#sc-4252AK, Santacruz Biotechnology, Dallas, TX, USA). Cells were harvested and washed with PBS. Then, they were resuspended in Annexin V binding buffer and separated into four groups (Annexin V, propidium iodide (PI), Annexin V + PI, and NC). Data were analyzed by using FACSCalibur (BD biosciences, Franklin Lakes, NJ, USA) flow cytometry.

### 4.4. Cell Cycle Analysis

Cells were seeded into T25 flasks at a density of 50 × 10^3^. The following day, treatments were applied and cells were further incubated for 72 h at 37 °C. Then, they were harvested and washed with PBS and fixed with 70% ice-cold ethanol for at least two hours at −20 °C. Cell pellets were permeabilized with 0.1% triton-X-100 and incubated with 20 μg/mL RNase at room temperature for 30 min. Finally, cells were stained with PI and then immediately analyzed by a 488 nm single laser emitting device within 15 min.

### 4.5. Real-Time PCR

Total RNA was isolated by using an RNA isolation kit (#740955.250, Macherey-NAGEL, Düren, Germany) according to the manufacturer’s instructions. RNA concentrations were determined by absorbance at 260 nm using a spectrophotometer. After that, cDNA was synthesized using QuantiTect Reverse Transcription Kit (#205313, QIAGEN, Hilden, Germany) according to the manufacturer’s protocols. RT-PCR was performed using SYBR Green (#4309155, Thermo Fisher, Waltham, MA, USA) and assayed in triplicate using the iCycler RT-PCR detection system (Bio-Rad, Hercules, CA, USA). The expression levels were normalized with respect to RPL30 (Ribosomal Protein L30) gene (F: 5′-ACAGCATGCGGAAAATACTAC-3′ R: 5′-AAAGGAAAATTTTGCAGGTTT-3′) levels. Genes and their corresponding primer sequences used in this study were as follows: Tumor protein 53 (TP53) (F: 5′-GCCCAACAACACCAGCTCCT-3′ R: 5′-CCTGGGCATCCTTGAGTTCC-3′) baculoviral inhibitor of apoptosis repeat-containing 5 (BIRC5 or Survivin) (F: 5′-TCTTCACCGCTTTGCTTTC-3′ R: 5′-CGCACTTTCTCCGCAGTTTC-3′), Bcl-2-associated X protein (BAX) (F: 5′-TGCAGAGGATGATTGCCGCCG-3′ R: 5′-ACCCAACCACCCTGGTGTTGG-3′), Tyrosine-protein kinase (ABL-1) (F: 5′-TACCCGATTGACCTGTC-3′ R: 5′-CGATTTCAGCAAACGACCCC-3′), proliferating cell nuclear antigen (PCNA) (F: 5′-CAAGTAATGTCGATAAAGAGGAGG-3′ R: 5′-GTGTCACCGTTGAAGAGAGTGG-3′) marker of proliferation Kiel 67 (Ki-67) (F: 5′-GAAAGAGTGGCAACCTGCCTTC 3′ R: 5′-GCACCAAGTTTTACTACATCTGCC 3′) E2F Transcription Factor 4 (E2F4) (F: 5′-GCATCCAGTGGAAGGGTGTG3′ R: 5′-ACGTTCCGGATGCTCTGCT3′) growth arrest and DNA damage inducible alpha (GADD45A) (F: 5′-GATGCCCTGGAGGAAGTGCT3′ R: 5′-GAGCCACATCTCTGTCGTCGT3′), Ataxia Telangiectasia Mutated (ATM) (F: 5′-TGTTCCAGGACACGAAGGGAGA-3′ R: 5′-CAGGGTTCTCAGCACTATGGGA-3′), ataxia-telangiectasia-and Rad3-related (ATR) (F: 5′-GGAGATTTCCTGAGCATGTTCGG-3′ R: 5′-GGCTTCTTTACTCCAGACCAATC-3′), B-cell lymphoma 2 (BCL-2) (F: 5′-AACGGAGGCTGGGATGCCTTTGTG-3′ R: 5′-ACCAGGGCCAAACTGAGCAGAGT-3′), Tafazzin (TAZ) (F: 5′-GGCTGGGAGATGACCTTCAC-3′ R: 5′-CTGAGTGGGGTGGTTCTGCT-3′), connective tissue growth factor (CTGF) (F: 5′-AGGAGTGGGTGTGTGACGA-3′ R: 5′-CCAGGCAGTTGGCTCTAATC-3′), Cysteine-rich angiogenic inducer 61 (CYR61) (F: 5′-CCTTGTGGACAGCCAGTGTA-3′ R: 5′-ACTTGGGCCGGTATTTCTTC-3′), B-Raf Proto-Oncogene, Serine/Threonine Kinase (BRAF) (F: 5′-CTATTCCACAAAGCCACAACTG-3′ R: 5′-CGTGTAAGTAATCCATGCCCT-3′) Kristen rat sarcoma viral oncogene homolog (KRAS) (F: 5′-TCCAACAATAGACGATTCCTACAG-3′ R: 5′-GCAAATCAAAGAAAGCCC-3′), and Kinetochore-associated protein-1 (KNTC-1) (F: 5′-ATAGTCAACCCAGAGTGGGCTGT-3′ R: 5′-TTTCACGTTTTTCGTGCTGCTGCG-3′). The fold changes for each sample were determined using the 2(−Delta Delta C(T)) method.

### 4.6. Scratch Assay

HCT-116 and HT-29 cancer cells were seeded in 6-well plates at 1 × 10^6^ cells/well and grown to greater than 90% confluency. Due to its semi-suspension growth characteristics, the COLO-205 cell line was excluded from this particular experiment. Cells were scratched with a sterile 200 µL pipette tip and then washed three times with PBS to remove cellular debris. Then, cells were cultured in complete medium, and IC-50 values of NaB were applied to HCT-116 and HT-29 cancer cell lines. After 0, 24, 48, and 72 h of incubation, images were taken using a light microscope.

### 4.7. Colony-Formation Assay

For the colony-formation assay, HCT-116 and HT-29 cells were seeded in a 6-well plate at a density of 500 cell/well. Due to its semi-suspension growth characteristics, the COLO-205 cell line was excluded from this particular experiment. After 24 h, the media were changed and cells were treated with IC-50 concentration of NaB. The cells were grown for 15 days at 37 °C and 5% humidified CO2 to form colonies. After 15 days, the cells were washed twice with PBS, fixed with 4% paraformaldehyde (PFA), and stained with 0.5% crystal violet for 15 min at room temperature. Then, the dye was washed with PBS from the wells, photos were taken with a luminometer device (Biorad, Hercules, CA, USA), and colonies were counted.

### 4.8. Caspase Activity Assay

Caspase activity in HCT116, HT-29, and Colo-205 cells was measured after NaB treatment of cells for 72 h by using Caspase-Glo^®^ 3/7, Caspase-Glo^®^ 8, and Caspase-Glo^®^ 9 assay systems (Promega, Madison, WI, USA) according to the manufacturer’s instructions. Shortly, cells were cultured in white 96-well plates, and the following day they were treated with NaB for 72 h. Caspase levels were measured from 0 min to 180 min by a luminometer.

### 4.9. Ethynyl-2′-Deoxyuridine Assay

EdU Staining Proliferation Kit (iFluor 647) (Abcam, Cambridge, MA, USA; ab222421) was applied as recommended by the manufacturer. HCT116, HT-29, and Colo-205 cells were seeded in 4 wells (Millicell^®^ EZ Slide, 4-well, Merck Millipore, Burlington, MA, USA), and after 72 h, cells were treated with a culture medium containing 20 μM EdU reagent. Next, cells were incubated for 2 h and were fixed with paraformaldehyde. Nuclei were stained with DAPI. Fluorescence images were taken with a confocal microscopy.

### 4.10. Immunocytochemistry (ICC)

HCT-116 and HT-29 cells were seeded in 4 wells (Millicell^®^ EZ Slide, 4-well), and after 72 h, the cells were fixed with 2% paraformaldehyde for 30 min at 4 °C. Due to its semi-suspension growth characteristics, the COLO-205 cell line was excluded from this particular experiment. After, 0.1% Triton-X100 was added to each well for 10 min to permeabilize cells at room temperature. A total of 10% FBS was added to each well to block cells. After that, cells were incubated with primary antibodies 1:1000 Phospho-YAP (ab105105), MST-1 (ab51134), and SAV-1 (ab230265) overnight. Then, cells were incubated with a secondary antibody (1:200) for 2 h. DAPI (1:1000) was added to each well for 20 min at 4 °C. The percentage of stained cells was quantified and analyzed using confocal microscopy.

### 4.11. Statistical Analysis

All data are shown as the means ± standard errors. The statistical analysis of the results was performed with a two-way ANOVA, and graphs were drawn using GraphPad Prism 5 software. Statistical significance was determined at ns: non-significant, *: *p* < 0.05, **: *p* < 0.01, ***: *p* < 0.001, and ****: *p* < 0.0001.

## 5. Conclusions

Collectively, our findings present strong evidence that NaB not only induces cell death in colorectal cancer cells via both intrinsic and extrinsic apoptotic pathways but also halts proliferative and migratory behaviors through G2/M cell cycle arrest and the inactivation of YAP/TAZ oncogenic signaling. These attributes make NaB a promising candidate for further development as a novel therapeutic agent, potentially used in combination with existing chemotherapeutics or as a part of targeted strategies aimed at critical molecular modulators like the Hippo pathway. Moving forward, further studies involving in vivo toxicity assessments, pharmacokinetics, and functional validation of molecular targets—particularly YAP/TAZ—will be critical to fully establish its translational potential in colorectal cancer therapy.

## Figures and Tables

**Table 1:** IC50 values of HCT-116, COLO-205, HT-29, and CCD-18CO cell lines for SPP treatment at 72 h.

IC-50 Values of Cell Lines	Treatment of NaB
HCT-116	1000 µg/mL
COLO-205	500 µg/mL
HT-29	500 µg/mL
CCD-18CO	2500 µg/mL

## Data Availability

Data is contained in the paper.

## Abbreviations

CRC	Colorectal cancer
NaB	Sodium Pentaborate Pentahydrate
FDA	Food and Drug Administration
LATS1/2	Large Tumor Suppressor Kinase 1/2
MST1/2	Mammalian STE20-like Kinase
SAV1	Salvador Homologue 1
MOB1A/B	MOB Kinase Activator 1 A
YAP	Yes-associated protein 1
TAZ	WW-domain-containing transcription regulator 1
TEAD	Transcriptional Enhanced Associated Domain
MTS	3-(4,5-dimethyl-thiazol-2)-5-(3-carboxy-methoxy-phenyl)-2-(4-sulfo-phenyl)-2H-tetrazolium salt
PFA	Paraformaldehyde
